# Anatomical drivers of organ size and trait coordinated association between pseudobulbs and roots in *Dendrobium*

**DOI:** 10.1186/s40529-026-00495-1

**Published:** 2026-06-24

**Authors:** Feng-Ping Zhang, Dong-Qin Lǚ, Han-Run Li, Shi-Bao Zhang

**Affiliations:** 1 College of Ethnic Medicine, Yunnan Key Laboratory of Dai and Yi Medicines, Yunnan University of Chinese Medicine, Kunming, Yunnan 650500 China; 2 Kunming Botanical Garden, Kunming Institute of Botany, Chinese Academy of Sciences, Kunming, Yunnan 650201 China; 3 Key Laboratory of Economic Plants and Biotechnology, Yunnan Key Laboratory for Wild Plant Resources, Kunming Institute of Botany, Chinese Academy of Sciences, Kunming, Yunnan 650201 China

**Keywords:** Anatomy, Pseudobulb, Root, Organ size, Trait coordination, Epiphytic orchids

## Abstract

**Background:**

Organ size is a key phenotypic trait linking plant structure, physiological function, and ecological adaptation. The epiphytic orchid genus *Dendrobium* comprises highly diversified species adapted to heterogeneous canopy microhabitats, where water and nutrients are scarce and unpredictable. It has evolved two specialized organs, water-storing pseudobulbs and absorptive succulent roots that are critical for epiphytic survival. However, their central role in drought adaptation, interspecific anatomical variation and the drivers of organ size remain poorly understood. Here, we quantified 7 pseudobulb and 13 root traits across 37 *Dendrobium* species, using phylogenetic independent contrasts to explore the relationships among traits.

**Results:**

All traits exhibited considerable interspecific variation, with pseudobulbs showing greater trait lability than roots, reflecting diversified water-use strategies under epiphytic pressure. Weak phylogenetic signals across traits indicate environmental selection dominates anatomical diversification. Pseudobulb radius correlated strongly with parenchyma area and vascular bundle traits, regardless of phylogenetic correction, but not with epidermis thickness after phylogenetic correction. Root radius was associated closely with cortex, velamen, and vascular bundle traits across analyses, but not with exodermis thickness after phylogenetic correction. Functionally analogous traits (pseudobulb radius vs. root radius; pseudobulb vascular bundle area vs. root vascular bundle area and root exodermis area; pseudobulb parenchyma area vs. root cortex thickness, velamen area, and velamen cell area) between pseudobulbs and roots were consistently and positively correlated, revealing coordinated water-use strategies across organs.

**Conclusion:**

*Dendrobium* organ size is determined by tissue-level adaptive architecture. Pseudobulb size depends primarily on water-storage parenchyma, while root size is tightly linked to absorptive velamen. The species-level variation, driven mainly by environmental selection, underscores diversified hydraulic strategies in epiphytic orchids. Coordinated trait association between pseudobulb and root traits reflects adaptive integration of storage and absorption organs, a key evolutionary strategy for epiphytic survival. These findings highlight the fundamental role of cell and tissue dimensions in shaping orchid organ morphology.

**Supplementary Information:**

The online version contains supplementary material available at 10.1186/s40529-026-00495-1.

## Background

Plant organ size is shaped by long-term evolutionary adaptation, representing a coordinated response of plants to both biotic and abiotic selective pressures in specific ecological niches (Mizukami 2001; Horiguchi and Tsukaya 2011; Powell and Lenhard 2012). It critically modulates key fitness-related traits, including resource acquisition, photosynthetic efficiency, reproductive output, and stress tolerance, thereby influencing the distribution and abundance of plant species across heterogeneous habitats (Dovrat et al. 2019; Bazmi and Panichayupakaranant 2023). Understanding the mechanisms governing organ size variation is therefore central to advancing knowledge in plant developmental biology, evolutionary ecology, and conservation biology, particularly for species with specialized morphological structures that enable adaptation to extreme or resource-limited environments.

The determination of plant organ size is a complex process governed by the interplay of cellular, anatomical, developmental, genomic, and environmental factors (Mizukami 2001; Inagaki and Umeda 2011; Gázquez and Beemster 2017; Ma et al. 2025). At the cellular level, organ size is primarily shaped by two core processes: cell division, which dictates cell number, and cell expansion, which regulates cell dimensions, including length, width, and area (Gázquez and Beemster 2017). These processes are tightly coordinated by developmental regulatory programs, including key regulatory genes, (e.g. cyclins controlling cell division cycles and expansins modulating cell wall loosening) and hormone signaling cascades that collectively shape tissue patterning (Inagaki and Umeda 2011; Cosgrove 2015; Li et al. 2025). Beyond cellular control, tissue allocation and anatomical architecture are increasingly recognized as pivotal determinants of organ size, as they establish the structural framework for resource storage, transport, and metabolism (Xu et al. 1998; Mir-Rosselló et al. 2025). For instance, in succulent plants, the size of storage organs is largely determined by the dimensions and volume of parenchyma cells, which are specialized for accumulating water and carbohydrates to cope with arid conditions (Griffiths and Males 2017), a pattern reflecting adaptive tissue allocation to optimize functional performance.

The genus *Dendrobium*, a highly diverse lineage of epiphytic orchids, has evolved two key specialized organs: pseudobulbs and succulent roots. These structures are critical for survival in canopy habitats, where water and nutrients are scarce and unpredictable (De and Biswas 2022). Pseudobulbs, modified succulent stems, function as the primary storage organs for water and carbohydrates, enabling plants to endure periods of drought and nutrient limitation (De and Biswas 2022). *Dendrobium* roots are equally specialized, featuring a multi-layered velamen, a unique epidermal tissue composed of dead, lignified cells with distinct dimensions that form a porous network for rapid water uptake and retention (Zotz and Winkler 2013; Qi et al. 2020). The size of pseudobulbs and roots is therefore expected to be tightly linked to the dimensions of these specialized cells and tissues, as well as their tissue allocation patterns, with variation reflecting adaptive responses to heterogeneous microenvironments (Rai et al. 2025).

Despite the ecological and evolutionary significance of pseudobulb and root size in *Dendrobium*, the mechanisms underlying their variation, particularly the contributions of anatomical architecture, tissue allocation, and developmental regulation remain poorly explored. Most previous studies on *Dendrobium* organ morphology have focused on descriptive anatomy, medicinal compound biosynthesis, or floral trait regulation, with limited attention to the quantitative contributions of cell dimensions and tissue allocation in shaping organ size variation (Li et al. 2021; Burzacka-Hinz et al. 2025; Wang et al. 2025; Zhou et al. 2025). Furthermore, few studies have accounted for phylogenetic relationship when investigating organ size determinants, leaving critical gaps in our understanding of how anatomical, developmental, and evolutionary processes jointly shape these specialized non-leaf organs. Additionally, few studies have explored correlations between functionally analogous traits in pseudobulbs and roots, which may reveal integrated adaptive strategies for coping with the unique challenge of epiphytic lifeform.

Pseudobulbs and roots exhibit highly specialized anatomical structures adapted for water storage and absorption, respectively. We investigated 37 *Dendrobium* species, quantified seven pseudobulb anatomical traits and 13 root anatomical traits across these taxa, and conducted phylogenetically independent contrast (PIC) analysis to disentangle adaptive variation from phylogenetic effects. Our specific objectives were: (1) to quantify interspecific anatomical variation in pseudobulb and root anatomical traits; (2) to identify the primary anatomical cell dimensional, and tissue-level determinants of pseudobulb and root size; and (3) to explore correlations between functionally analogous traits in pseudobulbs and roots, and to assess their implications for epiphytic adaptation. This study aims to provide novel insights into the structural, and developmental mechanisms underlying the development of specialized organs in epiphytic plants, with broader relevance to understanding organ size regulation in resource-limited habitats.

## Materials and methods

### Plant material

Thirty-seven cultivated *Dendrobium* species were selected as experimental materials for the present study (Table S1). All plant samples were collected from a greenhouse in Kunming Botanical Garden, Kunming Institute of Botany, Chinese Academy of Sciences (elevation 1990 m; 102°41′ E, 25°10′ N). The consistent cultivation practices were applied to minimize environmentally induced phenotypic plasticity and reduce confounding effects from variable microhabitats in the field, thereby enabling us to detect genetically based interspecific differences in anatomical traits. Prior to sampling, plants were cultivated under uniform conditions for three years and watered as needed to ensure full acclimation to the common environment.

### Anatomical trait measurement

To characterize the anatomical traits, 3–6 pseudobulbs were sampled and cut into 1 cm segments from their middle regions, while 3–6 aerial roots were segmented into 0.5 cm fragments. All segments were fixed in FAA fixation (composed of 37% formaldehyde, glacial acetic acid, 95% ethanol, and deionized water at a volume ratio of 10:5:50:35) at room temperature for 48 h, followed by dehydration through a graded ethanol series (70%, 80%, 90%, 95%, and 100% ethanol) with a 30-min incubation period for each concentration. The dehydrated samples were embedded in paraffin wax, and the paraffin-embedded blocks were sectioned into thin slices using a rotary microtome (Leica RM2126RT, Germany). The sections were mounted onto pre-coated glass slides, followed by dewaxing in xylene and gradient rehydration. Subsequently, the sections were stained with safranin. Transverse sections were observed, photographed, and analyzed under a light microscope (Olympus BX53) equipped with a digital camera (Olympus DP74). Seven pseudobulb and thirteen root anatomical traits were quantified and measured using ImageJ software. Their units and functional implications are summarized in Table 1.

**Table 1:** Variations in pseudobulb and root anatomical traits of the tested *Dendrobium* species

Organ	Traits	Abbreviation	Unit	Functional significance	Range	CV (%)	Mean ± SE
Pseudobulb	Radius	PR	mm	Water availability	1.17–5.75	38.35	2.66 ± 0.17
	Epidermis thickness	ET	µm	Water conservation	8.77–41.75	33.72	20.05 ± 1.11
	Number of vascular bundle	*N*_vas_	No.	Water transport	31.67–143.80	38.26	81.74 ± 5.14
	Area of individual vascular bundle	*A*_ivas_	mm^2^	Water transport	0.01–0.05	46.64	0.02 ± 0.002
	Area of vascular bundle	*A*_pvas_	mm^2^	Water transport	0.41–6.04	74.03	1.86 ± 0.23
	Area of parenchyma	*A*_par_	mm^2^	Water storage	4.90-103.14	83.29	25.45 ± 3.49
	Area of parenchyma cell	*A*_parc_	mm^2^ × 10^− 3^	Water storage	1.26–13.93	44.50	6.49 ± 0.47
Root	Radius	RR	mm	Water availability	0.47–1.21	22.20	0.74 ± 0.03
	Root cortex thickness	RCT	µm	Water storage	176.01-520.93	22.72	271.39 ± 10.14
	Layer of velamen	LV	No.	Water conservation	4.00-14.50	31.10	6.22 ± 0.32
	Velamen thickness	VT	mm	Water conservation	0.10–0.68	37.56	0.27 ± 0.02
	Area of velamen	*A*_vel_	mm^2^	Water conservation and storage	0.36–3.60	56.81	1.14 ± 0.11
	Velamen cell length	VCL	µm	Water storage	31.96–78.67	18.46	54.42 ± 1.65
	Velamen cell width	VCW	µm	Water storage	21.52–32.76	11.62	26.46 ± 0.51
	Area of velamen cell	*A*_velc_	mm^2^ × 10^− 3^	Water storage	0.69–2.41	27.11	1.50 ± 0.07
	Number of exodermis cell	*N*_exo_	No.	Water transport	91.50-232.67	19.10	132.53 ± 4.16
	Exodermis thickness	EXT	µm	Water transport	27.09-44.00	10.03	34.29 ± 0.57
	Area of exodermis	*A*_exo_	mm^2^	Water transport	0.06–0.19	26.07	0.10 ± 0.004
	Number of endodermis cell	*N*_end_	No.	Water transport	47.50-83.25	13.20	61.40 ± 1.33
	Area of vascular bundle	*A*_rvas_	mm^2^	Water transport	0.05–0.28	35.90	0.14 ± 0.008

### Statistical analysis

Regression analyses were performed to examine the trait correlations. We specifically evaluated the associations between pseudobulb radius and its anatomical traits, as well as between root radius and the corresponding root anatomical traits. To further assess the relative contribution of individual anatomical traits to the variations in pseudobulb and root radius, a hierarchical partitioning approach was applied using the R package “glmm.hp” (Lai et al. 2022, 2023). Pearson’s correlation analysis was conducted to quantify the pairwise correlations among all measured anatomical traits. The underlying molecular phylogeny was inferred from whole-genome re-sequencing data (Zhang et al. 2025). The phylogenetic tree was constructed based on single nucleotide polymorphisms (SNPs) using the Bayesian inference (BI) method, *Bletilla striata* was used as the outgroup to root the phylogenetic tree (Zhang et al. 2025). MrBayes 3.2.7a software (Ronquist and Huelsenbeck 2003) was employed for the BI analysis. This analysis involved two independent Markov Chain Monte Carlo (MCMC) runs, each consisting of two million generations (Zhang et al. 2025). Phylogenetic signals of all measured pseudobulb and root anatomical traits were tested using the K-statistic, which is based on a ‘Brownian motion model’ of trait evolution (Blomberg et al. 2003). The K-statistic was performed using the R package “picante”. Phylogenetically independent contrast (PIC) analysis was performed using the “ape” package to account for phylogenetic relationships among species to explore the evolutionary relationships between pseudobulb and root anatomical traits of *Dendrobium* and the biological strategy of coordinated evolution of different organs to adapt to water-deficient environments. All statistical analyses were performed in R software (v.4.4.2; R Core Team 2020).

## Results

### Variations and phylogenetic signals in anatomical traits of *Dendrobium* pseudobulbs and roots

**Fig. 1:**
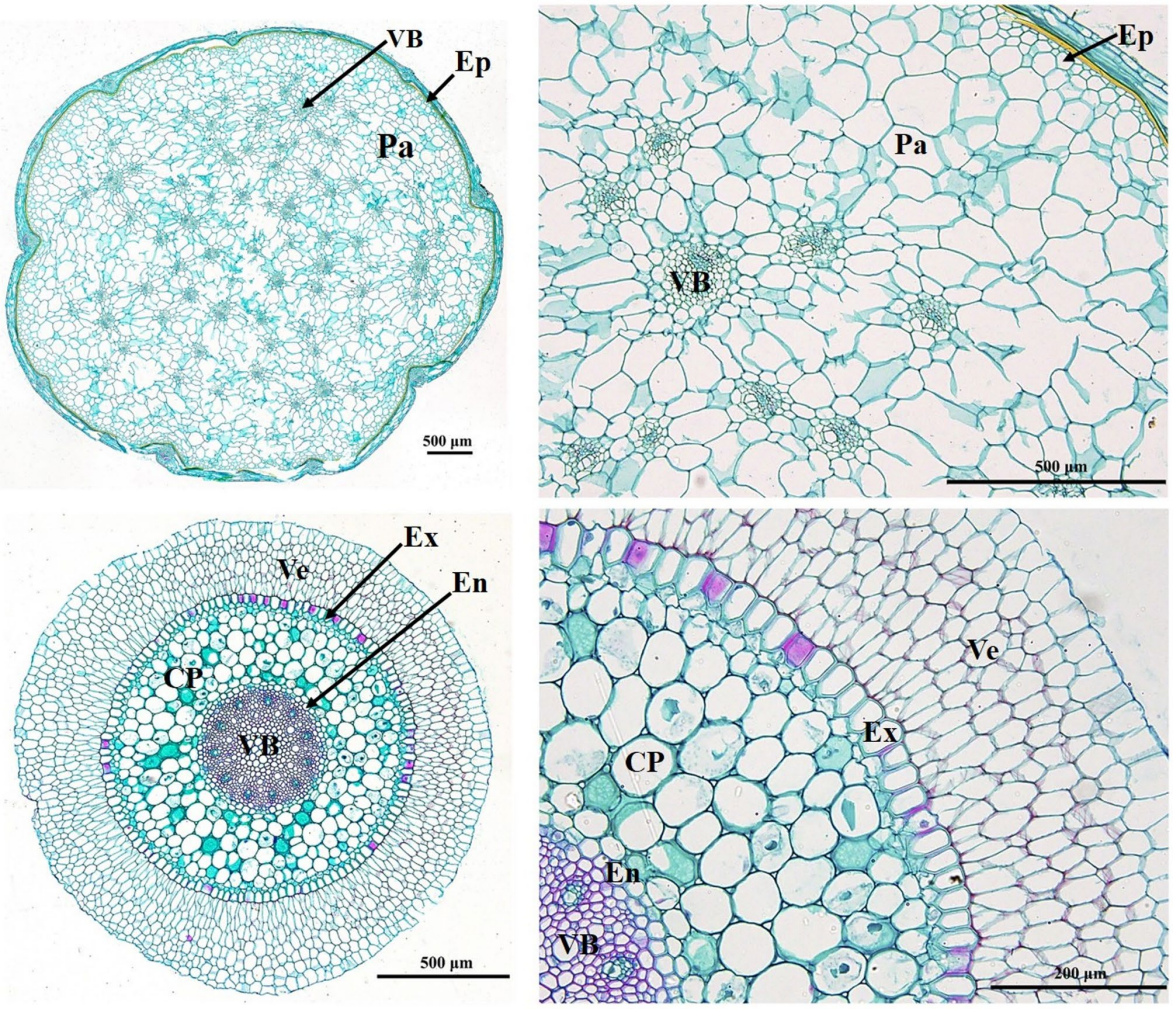
Comparative anatomical structures of pseudobulb and root in *Dendrobium officinale*. Ep, epidermis; Pa, parenchyma; VB, vascular bundle; Ve, velamen; Ex, exodermis; CP, cortical parenchyma; En, endodermis

As typical epiphytic orchids, *Dendrobium* rely on pseudobulbs for water and nutrient storage and roots for water uptake and mechanical fixation to survive heterogeneous canopy microhabitats. We quantified seven pseudobulb traits and 13 root traits across 37 *Dendrobium* species (Fig. 1), and detected high interspecific variation in all measured anatomical traits (Table 1). Overall, pseudobulb traits exhibited distinctly higher variability than root traits, reflecting divergent adaptive strategies of storage and absorptive functions. Pseudobulb parenchyma area (CV = 83.29%) and vascular bundle area (74.03%) showed the highest variation, while epidermis thickness displayed the lowest (33.72%). For root traits, velamen area (56.81%), velamen thickness (37.56%), and vascular bundle area (35.90%) varied most, whereas exodermis thickness showed the lowest variation (10.03%).

Phylogenetic signal analysis (Table S2) revealed that all measured functional traits exhibited weak phylogenetic signals (*K* < 1), demonstrating that environmental selection is the dominant force shaping phenotypic differentiation across *Dendrobium* species. Notably, pseudobulb epidermis thickness displayed a significant but weak phylogenetic signal (*K* = 0.842, *P* = 0.001).

### Anatomical determinants of pseudobulb and root size

**Fig. 2:**
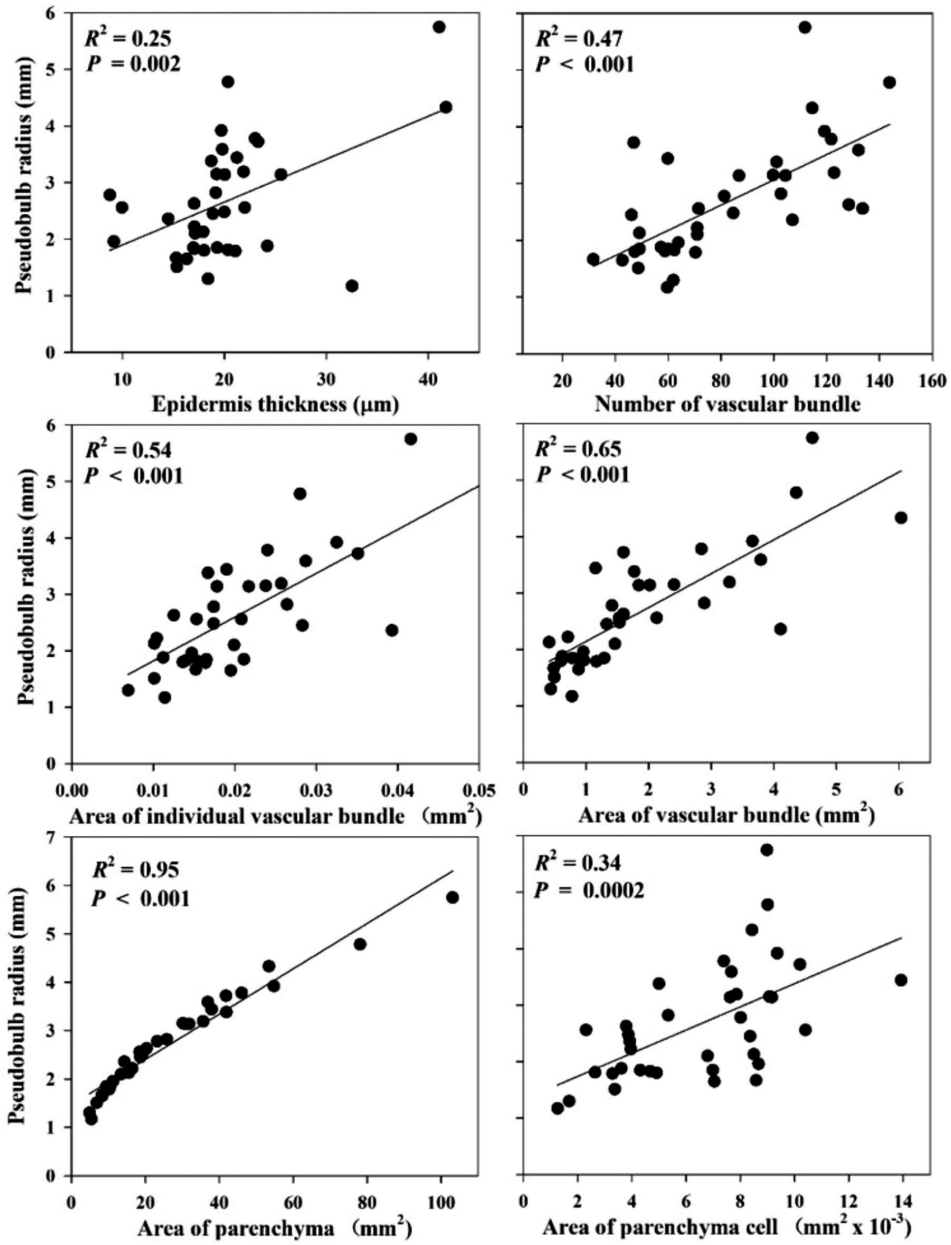
The relationships of pseudobulb radius with epidermis thickness, number of vascular bundle, area of individual vascular bundle, area of vascular bundle, area of parenchyma, and area of parenchyma cell in 37 *Dendrobium* species. Coefficient of determination (*R*²) and regression lines are shown in solid lines

**Fig. 3:**
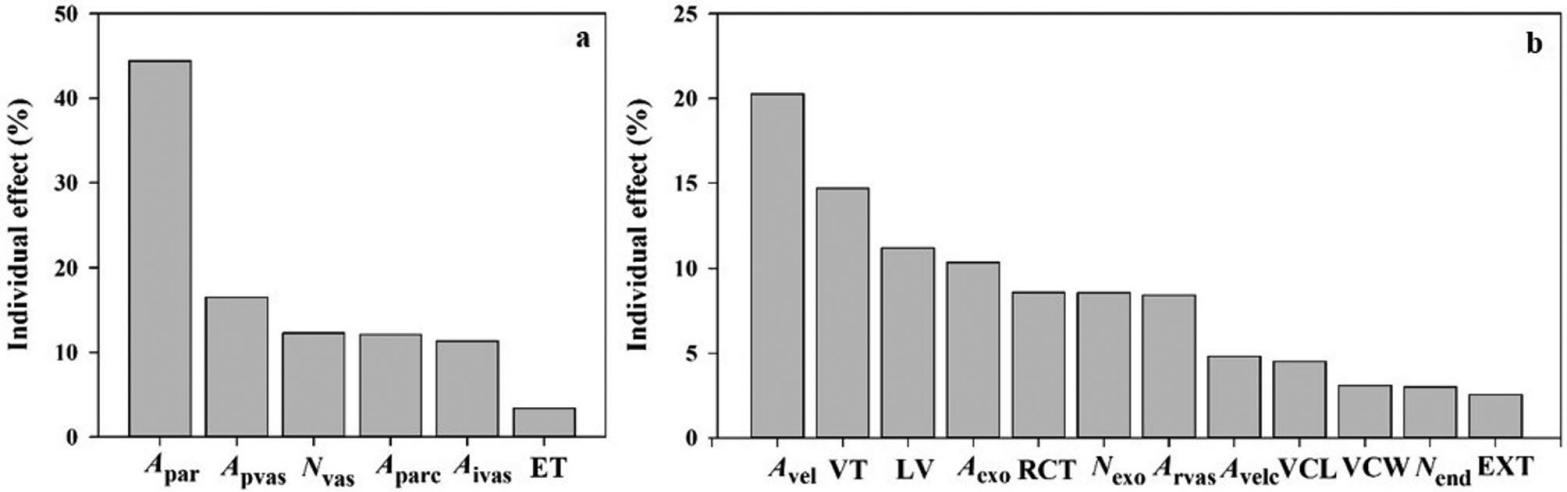
The relative importance of pseudobulb and root anatomical traits in explaining variances in pseudobulb radius (**a**) and root radius (**b**). Each bar shows variance explained by the independent effects of each variable. *A*_par_, area of parenchyma; *A*_pvas,_ area of vascular bundle; *N*_vas_, number of vascular bundle; *A*_parc_, area of parenchyma cell; *A*_ivas_, area of individual vascular bundle; ET, epidermis thickness; *A*_vel_, area of velamen; VT, velamen thickness; LV, layer of velamen; *A*_exo_, area of exodermis; RCT, root cortex thickness; *N*_exo_, number of exodermis cell; *A*_rvas_, area of vascular bundle; *A*_velc_, area of velamen cell; VCL, velamen cell length; VCW, velamen cell width; *N*_end_, number of endodermis cell; EXT, exodermis thickness

Pseudobulb radius was significantly and positively related to epidermis thickness (*R*² = 0.25, *P* = 0.002), number of vascular bundles (*R*² = 0.47, *P* < 0.001), area of individual vascular bundle (*R*² = 0.54, *P* < 0.001), total vascular bundle area (*R*² = 0.65, *P* < 0.001), parenchyma area (*R*² = 0.95, *P* < 0.001), and area of parenchyma cells (*R*² = 0.34, *P* = 0.0002) (Fig. 2). After accounting for phylogenetic effects using PICs, the relationship between epidermis thickness and pseudobulb radius became non-significant (*R*² = 0.03, *P* = 0.32) (Fig. S1). Among all predictors, parenchyma area contributed the most to pseudobulb size (44.40%) (Fig. 3).

**Fig. 4:**
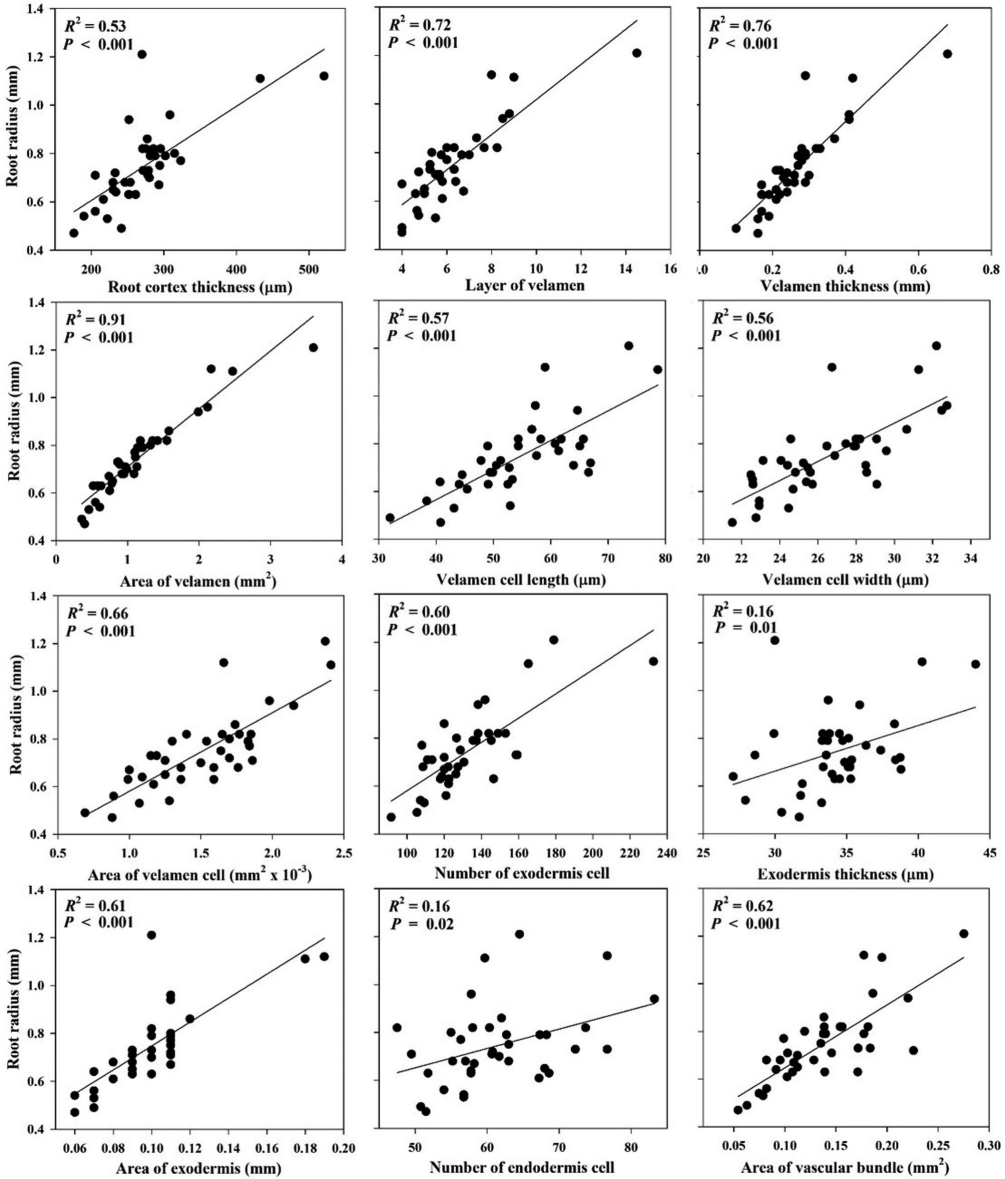
The relationships of root radius with root cortex thickness, layer of velamen, velamen thickness, area of velamen, velamen cell length, velamen cell width, area of velamen, number of exodermis cell, exodermis thickness, area of exodermis, number of endodermis cell, and area of vascular bundle in 37 *Dendrobium* species. Coefficient of determination (*R*²) and regression lines are shown in solid lines

Root radius was positively related to root cortex thickness (*R*² = 0.53, *P* < 0.001), number of velamen layers (*R*² = 0.72, *P* < 0.001), velamen thickness (*R*² = 0.76, *P* < 0.001), velamen area (*R*² = 0.91, *P* < 0.001), velamen cell length (*R*² = 0.57, *P* < 0.001), velamen cell width (*R*² = 0.56, *P* < 0.001), area of velamen cells (*R*² = 0.66, *P* < 0.001), number of exodermis cells (*R*² = 0.60, *P* < 0.001), exodermis thickness (*R*² = 0.16, *P* = 0.01), exodermis area (*R*² = 0.61, *P* < 0.001), number of endodermis cells (*R*² = 0.16, *P* = 0.02), and vascular bundle area (*R*² = 0.62, *P* = 0.003) (Fig. 4). After phylogenetic correction, root radius was not significantly related to exodermis thickness (*R*² = 0.06, *P* = 0.16) (Fig. S2). Velamen area made the largest contribution to root size (20.25%) (Fig. 3).

### Coordination between pseudobulb and root traits

**Fig. 5:**
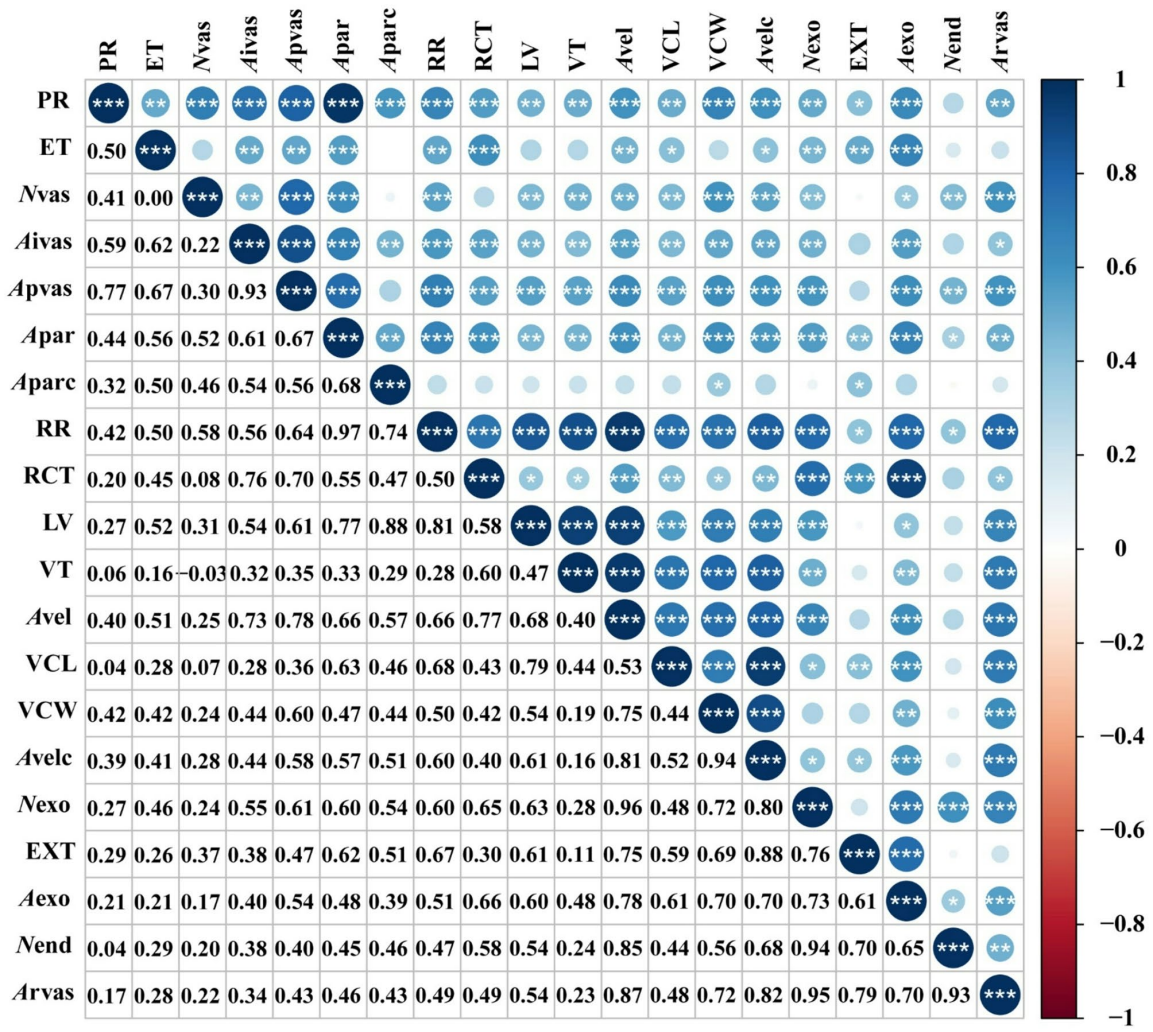
Pearson’s correlation coefficient analysis among pseudobulb radius, root radius, and anatomical traits in 37 *Dendrobium* species. Circle sizes and colors represent the significance and correlation coefficient (*r*). Significant levels are shown. *P < 0.05; **P < 0.01; ***P < 0.001. PR, pseudobulb radius; ET, epidermis thickness; *N*_vas_, number of vascular bundle; *A*_ivas_, area of individual vascular bundle; *A*_pvas,_ area of vascular bundle; *A*_par_, area of parenchyma; *A*_parc_, area of parenchyma cell; RR, root radius; RCT, root cortex thickness; LV, layer of velamen; VT, Velamen thickness; *A*_vel_, area of velamen; VCL, velamen cell length; VCW, velamen cell width; *A*_velc_, area of velamen cell; *N*_exo_, number of exodermis cell; EXT, exodermis thickness; *A*_exo_, area of exodermis; *N*_end_, number of endodermis cell; *A*_rvas_, area of vascular bundle

Significant correlations were detected between pseudobulb and root traits (Fig. 5, Fig. S3). Pseudobulb radius was positively correlated with root radius, regardless of phylogenetic correction. The consistent association across original and PIC datasets excludes phylogenetic interference and verifies adaptive coordination between the storage organ (pseudobulb) and absorptive organ (root). As the most prominent finding of this study, this cross-organ correlation reveals a long-term evolved functional syndrome across *Dendrobium* species. Such coordinated size variation effectively matches water storage capacity and water acquisition efficiency under variable epiphytic conditions. After phylogenetic correction, pseudobulb vascular bundle area was positively correlated with root vascular bundle area and root exodermis area. Pseudobulb parenchyma area remained significantly correlated with root cortex thickness, velamen area, and velamen cell area regardless of phylogenetic correction.

## Discussion

Organ size is a pivotal phenotypic trait that reflects plant structure, function, and ecological adaptation, acting as a core indicator of fitness across heterogeneous habitats (Dovrat et al. 2019; Bazmi and Panichayupakaranant 2023). For epiphytic *Dendrobium*, pseudobulbs and roots are specialized for water storage and uptake, critical for canopy survival (Benzing 1990; Zotz and Winkler 2013; Sabu and Paulose 2025). Here, using broad sampling across *Dendrobium* 37 species, we demonstrate that interspecific anatomical variation is predominantly shaped by environmental selection, with weak phylogenetic constraint, and driven by adaptive tissue allocation, an evolutionary strategy central to epiphytic water-use diversification.

### Variations and phylogenetic signals in anatomical traits of pseudobulbs and roots: Linked to tissue allocation patterns and ecological adaptation

This study revealed substantial interspecific variation in all tested anatomical traits of pseudobulbs and roots, with pseudobulb traits exhibiting greater variation than root traits. This pattern accords with recent evidence that roots exhibit extreme structural and hydraulic variability across species, including divergent vulnerability to xylem cavitation, contrasting stele anatomy, and tissue shrinkage dynamics (Harrison Day et al. 2023), highlighting roots as inherently variable organs. This divergent pattern of variability is tightly associated with the contrasting tissue allocation patterns and functional specialization of these two organs, which jointly support the epiphytic lifeform of *Dendrobium*. Given the unpredictable rainfall and limited nutrients in the canopy, pseudobulbs have evolved as primary reservoirs of water and non-structural carbohydrates, and their tissue allocation is optimized for storage function, thereby buffering against resource fluctuations (Zotz and Hietz 2001). The extreme variation of pseudobulb parenchyma and vascular tissues represents adaptive divergence in tissue allocation, which enables species to cope with diverse microhabitat conditions. In general, pseudobulbs possess high lability to adjust storage capacity in response to fluctuating resources, while anatomical structures remain functionally conserved to sustain stable water absorption, fixation and mechanical support.

Our results indicate that pseudobulb parenchyma area is the dominant and robust driver of organ size, representing the most variable and adaptively significant tissue in this study. The extreme variability in parenchyma area likely reflects adaptive diversification in storage tissue allocation across *Dendrobium* species. Weak phylogenetic signals confirm environmental selection dominates trait evolution, while only epidermis thickness shows mild constraint. This pattern highlights that species-level variation reflects adaptive radiation rather than phylogenetic conservatism, exploring future partitioning by clade, habitat, or rainfall to explore broader evolutionary ramifications. This key finding provides strong empirical support for the central hypothesis that tissue allocation strategy evolves in response to habitat moisture availability. Species inhabiting drier microhabitats may evolve a larger allocation of large-volume parenchyma cells to enhance water and carbohydrate reserves, enabling them to withstand prolonged drought periods; conversely, taxa in more humid environments may prioritize the allocation of vascular tissue to improve transport efficiency (Sun et al. 2020). This finding highlights adaptive tissue allocation prioritizing water storage, a critical trait for *Dendrobium* inhabiting dry epiphytic microhabitats. This observed pattern supports the resource availability hypothesis, which suggests that traits associated with resource storage exhibit heightened variability in heterogeneous environments to maximize fitness across diverse ecological niches (De and Biswas 2022).

Root traits displayed relatively low variability, particularly exodermis thickness, indicating strong functional conservation for protection and water retention. Orchid roots are specialized for dual functions: anchoring the plant to host substrates and facilitating rapid water absorption during transient rainfall events. The exodermis, in particular, forms a critical protective barrier against water loss and pathogen invasion (Joca et al. 2017; Sabu and Raji 2024). This differential variability strategy thus balances the need for flexibility in resource storage (pseudobulbs) with the requirement for functional stability in essential survival processes (roots), highlighting the adaptive significance of trait variation in epiphytic plants. Despite lower overall variability than pseudobulbs, the anatomical diversification observed in *Dendrobium* roots still conforms to the broader pattern that root systems exhibit pronounced structural lability (Harrison Day et al. 2023). Phylogenetic signal analysis revealed that all measured functional traits exhibited weak phylogenetic signals (*K* < 1), demonstrating that environmental selection dominates trait variation in *Dendrobium*.

### Anatomical determinants underlying pseudobulb and root size variation in *Dendrobium*: modulated by developmental regulation

Our results demonstrate that pseudobulb size was primarily governed by anatomical traits related to cell dimensions, with these traits being the direct phenotypic output of coordinated development regulation of cell proliferation and enlargement. A strong positive relationship was detected between pseudobulb radius and parenchyma area, with parenchyma area emerging as the dominant driver of size variation. Additionally, pseudobulb radius was significantly related to parenchyma cell area, indicating that both cell size and cell number, two key traits modulated by developmental signaling pathway, contribute to pseudobulb size modulation. Parenchyma tissue in *Dendrobium* pseudobulbs is composed of large, vacuolated cells specialized for water and carbohydrate storage (Sabu and Paulose 2025), and the development of these cells is regulated by core plant hormone signaling cascades (e.g., auxin, ABA, and cytokinin) (Li et al. 2025) and cell wall modification genes (e.g., expansins, and XTHs) (Qi et al. 2025). These developmental regulators modulate cell wall relaxation and vacuolar expansion, thereby determining parenchyma cell dimensions and, ultimately, the storage capacity and size of the pseudobulb. The dominant influence of parenchyma tissue on pseudobulb size, thus underscores the tight coupling between tissue structure, development regulation, and organ dimensions in specialized storage structures.

This finding aligns with previous studies on other plant storage organs. For instance, in potato tubers, parenchyma volume regulated by developmental pathways controlling cell size and proliferation, is identified as the primary determinant of organ size due to its central role in resource accumulation (Xu et al. 1998). Our study extends these insights by demonstrating that this link between developmental regulation of cell traits and organ size is conserved in epiphytic orchids, which face unique selective pressures such as fluctuating water supply and limited nutrient access. Furthermore, significant correlations were observed between pseudobulb diameter and vascular bundle traits, highlighting the integrated developmental regulation of storage and transport functions within this specialized organ. Vascular bundles facilitate the transport of stored resources to other organs (e.g., leaves, flowers), and their correlation with pseudobulb size suggests that the developmental programs governing storage tissue formation are coordinated with those regulation vascular tissue development to optimize resource utilization (De and Biswas 2022). This functional integration is critical for epiphytic plants, as it ensures that stored resources can be rapidly mobilized to support growth and reproduction when environmental conditions become favorable.

For roots, our results indicate that velamen traits closely associated with cell dimensions and their developmental regulation, are the key determinants of root diameter. Root radius was most strongly related with velamen thickness and velamen area; notably, significant positive correlations were also detected between root radius and velamen cell length, width, and area, modulated by developmental pathways, directly shape velamen structure and, consequently, root size. The velamen is a unique, multi-layered epidermal tissue in orchid roots, consisting of dead, lignified cells that form a porous network capable of rapidly absorbing and retaining water (Joca et al. 2017; Roth-Nebelsick et al. 2017). The development of the velamen is tightly regulated by epidermal cell fate specification and cell expansion pathways, and the tight correlation between velamen dimensions and root size in *Dendrobium* underscores the role of developmental regulation in shaping adaptive tissue structure and organ size in the epiphytic niche, where efficient capture and retention of limited water during short rainfall events is critical for survival.

Additionally, root radius was positively correlated with cortex thickness and total vascular bundle area, reflecting the integrated developmental regulation of three core root functions: water absorption (velamen), water storage (cortex), and resource transport (vascular bundles). The cortex of orchid roots contains parenchyma cells that store water and nutrients absorbed by the velamen, while vascular bundles facilitate the transport of these resources to the rest of the plant (De and Biswas 2022; Zhang et al. 2023), and the development of these tissues is coordinated by a shared set of developmental regulators to ensure functional integration. Similar patterns have been reported in epiphytic bromeliads, where root size correlates with traits related to water absorption and storage, indicating convergent evolution of developmental regulatory programs shaping root structure across phylogenetically distinct epiphytic lineages (Takahashi and Mercier 2024). Such convergence underscores the strong selective pressure exerted by resource limitation on the evolution of developmental pathways that govern organ structure and size in epiphytic plants.

### Association functionally between pseudobulb and root traits in *Dendrobium*: whole-plant coordination shaped by anatomical, developmental and genomic features

Functional coordination between pseudobulb and root traits represents a key evolutionary insight of this study. We detected significant positive correlations between functionally analogous traits in pseudobulbs and roots, reflecting integrated whole-plant adaptation to epiphytic habitats, an adaptation shaped by the joint action of anatomical architecture, developmental regulation, and underlying genomic features (Sun et al. 2020; Weigelt et al. 2021). For instance, pseudobulb radius was positively correlated with root radius, and this association persisted after phylogenetic correction, indicating an evolved adaptive syndrome of coordinated scaling between storage and absorption organs. Additionally, pseudobulb vascular bundle area correlated positively with root vascular bundle area. Most strikingly, pseudobulb parenchyma area exhibited a strong positive correlation with root velamen area. These relationships support the whole-plant functional coordination hypothesis, which posits that plants have evolve integrated trait syndromes across organs to maximize fitness under limiting environments (Qi et al. 2020; Liu et al. 2023).

In epiphytic *Dendrobium*, this coordination is critical for coping with the unpredictable availability of water and nutrients (De and Biswas 2022), as it may ensure that resource storage capacity (pseudobulbs) is matched to resource acquisition ability (roots) via the integration of anatomical traits, developmental programs, and genomic regulation. Combined with habitat-related tissue allocation patterns, this whole-plant coordination enables species to form complete adaptive strategies: dry-habitat species combine large storage pseudobulbs and highly developed absorptive roots to resist drought, while humid-habitat species coordinate relatively small storage organs and transport-oriented tissues to improve resource circulation. This pattern of inter-organ coordination has been documented in other epiphytic plants, such as ferns, where leaf and root traits are highly correlated and form a plant economics spectrum (Li et al. 2022). In *Dendrobium*, these correlations further highlight the unique adaptive strategies of orchids, which rely on specialized organs to survive in challenging canopy environments, and the genomic basis of this coordination is likely to involve cis-regulatory elements and transcription factors that control organ-wide developmental programs, rather than single gene changes. Furthermore, the strong positive relationships between functionally analogous traits suggest that evolutionary changes in the developmental regulation of one organ are likely to be accompanied by corresponding changes in the other, limiting the independent evolution of these traits and shaping the evolutionary trajectory of *Dendrobium* species (Qi et al. 2020). This evolutionary constraint may be ultimately encoded in the genome, with genomic features such as gene family expansion, regulatory network rewiring, and epigenetic modification shaping the developmental potential of different organs and their coordinated adaptation to the epiphytic niche.

## Conclusion

Our work reveals that organ size in epiphytic *Dendrobium* is not merely shaped by developmental anatomy, but driven by environment-mediated adaptive tissue allocation and whole-plant functional coordination, representing a key strategy in epiphytic water-use evolution. Specifically, pseudobulb size is governed by water-storing parenchyma, while root size depends on absorptive velamen. Weak phylogenetic signals confirm environmental selection as the primary driver of diversification, while conserved cross-organ correlations reveal an evolved adaptive syndrome that balances water storage and acquisition. This study establishes a general framework for understanding functional diversification in epiphytic plants.

## Supplementary Information

Below is the link to the electronic supplementary material.

Supplementary Material 1

## Data Availability

All data has been provided with the manuscript.
