# Supplementary material for: Anatomical drivers of organ size and trait coordinated association between pseudobulbs and roots in Dendrobium
Source: Bot Stud. 2026 Jun 24;67:8. doi: 10.1186/s40529-026-00495-1 (PMC13294433; doi:10.1186/s40529-026-00495-1)

**Supporting information**

**Table S1** The studied 37 *Dendrobium* species in this study.

| Species | Altitude (m) | Flowering period |
| --- | --- | --- |
| *D*. *aduncum* | 700-1000 | May-June |
| *D*. *aphyllum* | 400-1500 | March-April |
| *D*. *brymerianum* | 1100-1900 | June-July |
| *D. cariniferum* | 1100-1700 | March-April |
| *D. christyanum* | 800-1200 | June-July |
| *D. chrysanthum* | 700-2500 | September-October |
| *D. chrysotoxum* | 520-1260 | March-May |
| *D. crepidatum* | 1000-1800 | March-April |
| *D. crystallinum* | 540-1700 | May-July |
| *D. densiflorum* | 420-1000 | April-May |
| *D. devonianum* | 1850 | April-May |
| *D. falconeri* | 800-1900 | May-June |
| *D. fimbriatum* | 600-1700 | April-June |
| *D. findlayanum* | 800-900 | March |
| *D. flexicaule* | 1200-2000 | May |
| *D. gratiosissimum* | 800-1700 | April-May |
| *D. hancockii* | 700-1500 | May-June |
| *D. harveyanum* | 1100-1700 | March-April |
| *D. henryi* | 600-1700 | June-Septermber |
| *D. hercoglossum* | 590-1260 | May-June |
| *D. jenkinsii* | 700-1300 | April-May |
| *D. loddigesii* | 400-1500 | April-May |
| *D. lohohense* | 980-1 500 | June |
| *D. longicornu* | 1200-2500 | September-November |
| *D. moniliforme* | 1000-1300 | May |
| *D. moschatum* | 1300 | April-June |
| *D. nobile* | 480-1700 | April-May |
| *D. officinale* | 1600 | March-June |
| *D. parishii* | 43-201.3 | May-June |
| *D. pendulum* | 1050-1600 | March-April |
| *D. polyanthum* | 700-1800 | March-April |
| *D. sulcatum* | 700-800 | June |
| *D. stuposum* | 1800 | June |
| *D. thyrsiflorum* | 1100-1800 | April-May |
| *D. trigonopus* | 1150-1600 | March-April |
| *D. wardianum* | 1350-1900 | March-May |
| *D. wattii* | 2000 | August-November |

**Table S2.** Phylogenetic signals of pseudobulb and root anatomical traits in 37 *Dendrobium* species. Asterisks denote significant levels: ***P* < 0.01.

|  | Phylogenetic signal | |
| --- | --- | --- |
| Traits | *K* | *P* |
| PR | 0.345 | 0.046 |
| ET | 0.842 | 0.001** |
| *N*_vas_ | 0.121 | 0.598 |
| *A*_ivas_ | 0.342 | 0.083 |
| *A*_pvas_ | 0.189 | 0.425 |
| Apar | 0.35 | 0.071 |
| Aparc | 0.129 | 0.592 |
| RR | 0.159 | 0.576 |
| RCT | 0.139 | 0.709 |
| LV | 0.261 | 0.314 |
| VT | 0.251 | 0.334 |
| Avel | 0.272 | 0.293 |
| VCL | 0.140 | 0.625 |
| VCW | 0.131 | 0.606 |
| Avelc | 0.127 | 0.674 |
| Nexo | 0.180 | 0.487 |
| EXT | 0.141 | 0.570 |
| Aexo | 0.132 | 0.706 |
| Nend | 0.090 | 0.795 |
| Arvas | 0.116 | 0.712 |

Notes: PR, pseudobulb radius; ET, epidermis thickness; *N*_vas_, number of vascular bundle; *A*_ivas_, area of individual vascular bundle; *A*_pvas,_ area of vascular bundle; *A*_par_, area of parenchyma; *A*_parc_, area of parenchyma cell; RR, root radius; RCT, root cortex thickness; LV, layer of velamen; VT, Velamen thickness; *A*_vel_, area of velamen; VCL, velamen cell length; VCW, velamen cell width; *A*_velc_, area of velamen cell; *N*_exo_, number of exodermis cell; EXT, exodermis thickness; *A*_exo_, area of exodermis; *N*_end_, number of endodermis cell; *A*_rvas_, area of vascular bundle.

**Figure S1** Bivariate phylogenetic independent contrast relationships of pseudobulb radius with epidermis thickness, number of vascular bundle, area of individual vascular bundle, area of vascular bundle, area of parenchyma, and area of parenchyma cell in 37 *Dendrobium* species. Coefficient of determination (*R*²) and regression lines are shown in solid lines.


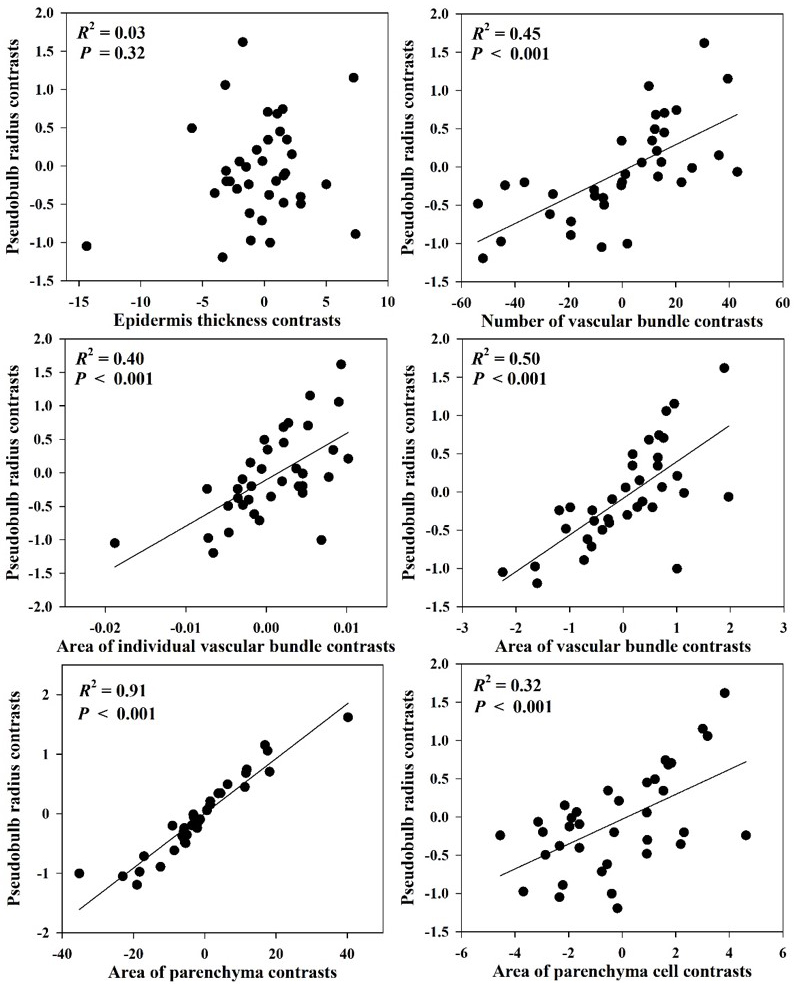


**Figure S2** Bivariate phylogenetic independent contrast relationships of root radius with root cortex thickness, layer of velamen, velamen thickness, area of velamen, velamen cell length, velamen cell width, area of velamen, number of exodermis cell, exodermis thickness, area of exodermis, number of endodermis cell, and area of vascular bundle in 37 *Dendrobium* species. Coefficient of determination (*R*²) and regression lines are shown in solid lines.


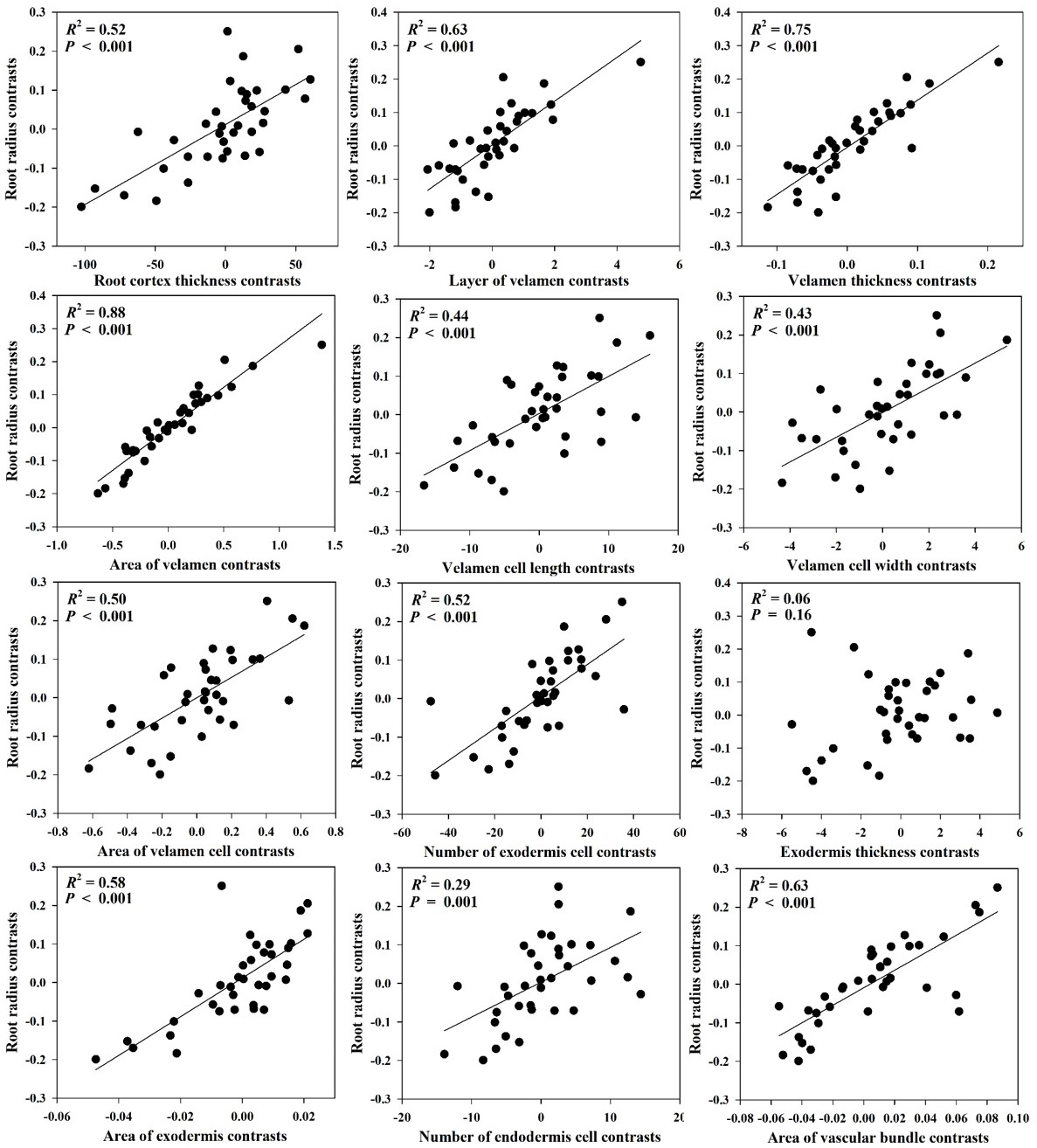


**Figure S3** Phylogenetic independent contrast correlations among pseudobulb radius, root radius, and anatomical traits in 37 *Dendrobium* species. Circle sizes and colors represent the significance and correlation coefficient (*r*). Significant levels are shown. **P* < 0.05; ***P* < 0.01; ****P* < 0.001. PR, pseudobulb radius; ET, epidermis thickness; *N*_vas_, number of vascular bundle; *A*_ivas_, area of individual vascular bundle; *A*_pvas,_ area of vascular bundle; *A*_par_, area of parenchyma; *A*_parc_, area of parenchyma cell; RR, root radius; RCT, root cortex thickness; LV, layer of velamen; VT, Velamen thickness; *A*_vel_, area of velamen; VCL, velamen cell length; VCW, velamen cell width; *A*_velc_, area of velamen cell; *N*_exo_, number of exodermis cell; EXT, exodermis thickness; *A*_exo_, area of exodermis; *N*_end_, number of endodermis cell; *A*_rvas_, area of vascular bundle.


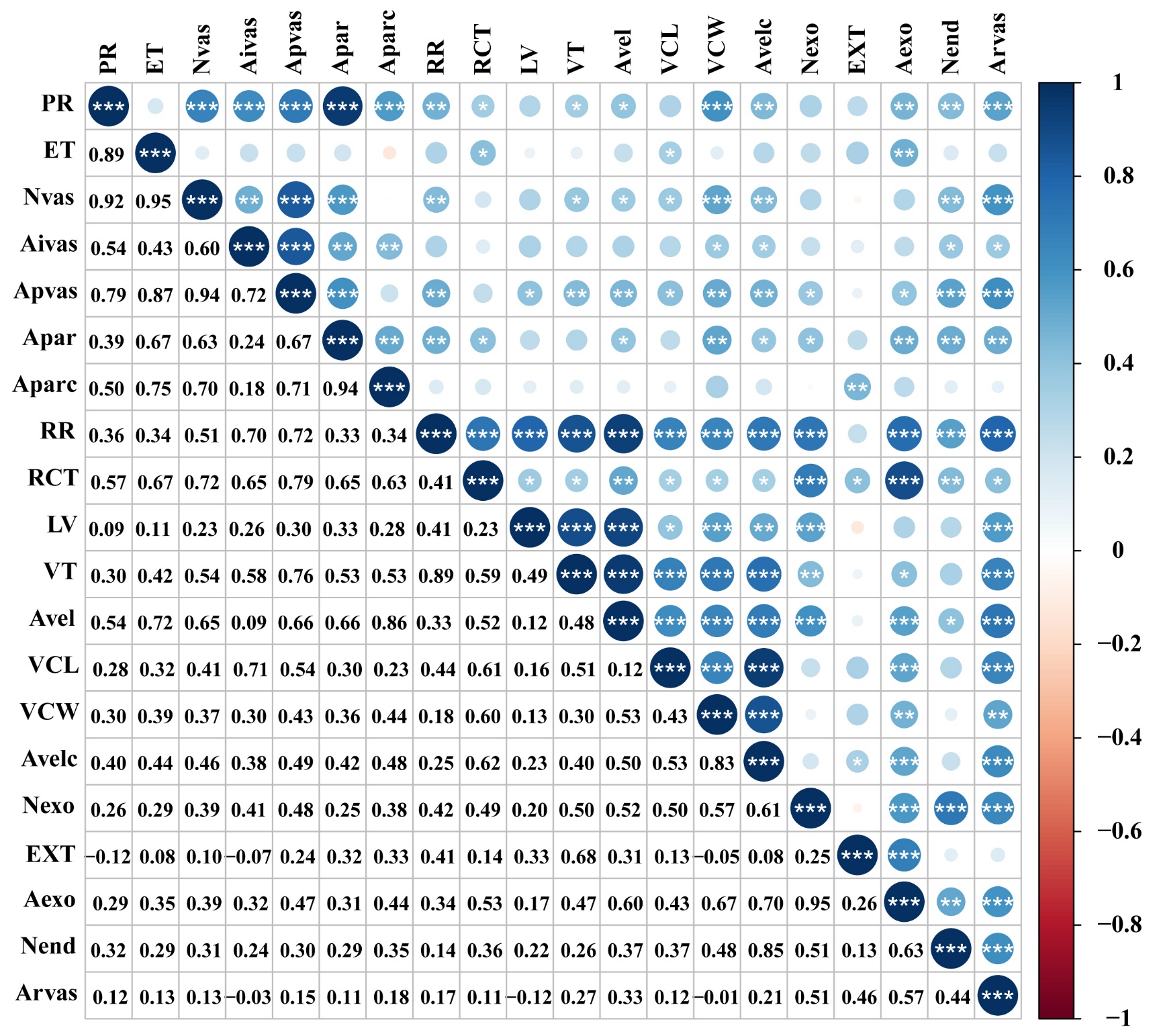

Supplement: Supplementary file 1 — Supplementary Material 1 [file 40529_2026_495_MOESM1_ESM.docx]
